# Low Obesity-Related Indices Are Associated with a Low Baseline Calcaneus Ultrasound T-Score, and a Rapid Decline in T-Score in a Large Taiwanese Population Follow-Up Study

**DOI:** 10.3390/nu15030605

**Published:** 2023-01-24

**Authors:** Li-Han Chen, Yi-Hsueh Liu, Szu-Chia Chen, Ho-Ming Su

**Affiliations:** 1 Graduate Institute of Clinical Medicine, College of Medicine, Kaohsiung Medical University, Kaohsiung 807, Taiwan; 2 Division of Cardiology, Department of Internal Medicine, Kaohsiung Medical University Hospital, Kaohsiung Medical University, Kaohsiung 807, Taiwan; 3 Department of Internal Medicine, Kaohsiung Municipal Siaogang Hospital, Kaohsiung Medical University Hospital, Kaohsiung Medical University, Kaohsiung 812, Taiwan; 4 Division of Nephrology, Department of Internal Medicine, Kaohsiung Medical University Hospital, Kaohsiung Medical University, Kaohsiung 807, Taiwan; 5 Faculty of Medicine, College of Medicine, Kaohsiung Medical University, Kaohsiung 807, Taiwan; 6 Research Center for Precision Environmental Medicine, Kaohsiung Medical University, Kaohsiung 807, Taiwan

**Keywords:** calcaneus ultrasound T-score, obesity-related indices, Taiwan biobank, follow-up

## Abstract

Osteoporosis results in reduced bone strength and an elevated risk of fractures. Both overweight and underweight have been associated with osteoporosis; however, few studies have examined associations between osteoporosis and indices related to obesity. Therefore, the aim of this study was to investigate the associations of obesity-related indices, including body mass index (BMI), waist–hip ratio (WHR), waist-to-height ratio (WHtR), body roundness index (BRI), body adiposity index (BAI), abdominal volume index (AVI), lipid accumulation product (LAP), and visceral adiposity index (VAI), with baseline and change in calcaneus ultrasound T-score between baseline and follow-up (ΔT-score). T-score was measured using ultrasound. A total of 26,983 subjects were enrolled (mean age 51.2 ± 10.4 years). Multivariable analysis showed significant associations between low BMI (per 1 kg/m^2^; β, 0.065), WHR (per 1%; β, 0.012), WHtR (per 1%; β, 0.024), BRI (per 1; β, 0.079), BAI (per 1; β, 0.032), AVI (per 1; β, 0.049), and LAP (per 1; β, 0.005) with low baseline T-scores (all *p* < 0.001). Furthermore, there were significant associations between low BMI (per 1 kg/m^2^; β, 0.005; *p* = 0.036), BAI (per 1; β, 0.010; *p* < 0.001), and VAI (per 1; β, 0.017; *p* = 0.002) with low ΔT-scores. A low baseline T-score was significantly associated with low values of LAP, AVI, BAI, BMI, BRI, WHR, and WHtR but not VAI. In addition, low BMI, BAI, and VAI were significantly associated with low ΔT-scores, representing a rapidly decreasing T-score. Consequently, avoiding being underweight may help prevent osteoporosis in the Taiwanese population.

## 1. Introduction

Osteoporosis is a serious health issue worldwide, characterized by reduced bone mass, disturbance of the microarchitecture, and skeletal fragility, leading to a reduction in bone strength and elevated fracture risk [1]. The presence of fragility fractures, particularly of the spine, wrist, hip, humerus, rib, and pelvis or T-scores ≤ −2.5 standard deviations at any site based on bone mineral density (BMD) measurements using dual-energy X-ray absorptiometry (DXA), can be used to make a clinical diagnosis of osteoporosis [2]. Known risk factors for osteoporosis included being overweight, smoking tobacco, excess alcohol intake, insufficient physical activity, malnutrition, and certain medications such as long-term steroid therapy [3]. Known non-modifiable risk factors include sex, age, race, and genetic characteristics [4]. The social and economic burden of osteoporosis is steadily increasing because of the aging global population. According to the United States health care system, osteoporotic fractures cost approximately USD 17 billion annually, a figure which is expected to approach USD 50 billion by 2040 [5]. Fractures and their clinical sequelae, including pain, disability, and death, are the main complications of osteoporosis [6]. Hip fractures have been associated with a 15–20% increase in mortality within 1 year, and a 2.5-fold increased risk of future fractures [6]. The disability resulting from fractures causes a heavy burden on society [7], and consequently, determining the risk factors for osteoporosis in order to decrease the prevalence of osteoporosis-related fractures is an important topic. Hence, early detection, diagnosis, and treatment of osteoporosis would improve patient treatment prognosis and quality of life. Therefore, we need indices to help us to diagnose osteoporosis early.

Many simple and conveniently obtained anthropometric indicators have been introduced to assess the central obesity-related risk of metabolic syndrome, including body mass index (BMI), waist–hip ratio (WHR), waist-to-height ratio (WHtR), body roundness index (BRI), body adiposity index (BAI), abdominal volume index (AVI), lipid accumulation product (LAP), and visceral adiposity index (VAI) [8,9]. These indicators can be calculated using routinely obtained parameters such as body weight (BW) and height (BH), waist (WC) and hip circumference (HC), triglycerides (TG), and high-density lipoprotein cholesterol (HDL-C). These obesity-related indices can evaluate obesity, which is defined by an excess accumulation of adipose tissue. Previous research has suggested the relationship between obesity with insulin resistance and type 2 diabetes, which is that nonesterified fatty acids secreted from adipose tissue in obese people may lead to insulin resistance and β-cell dysfunction [10]. Prior studies revealed that these obesity-related indices are predictors of hypertension [11,12,13]. Our recent research revealed that these obesity indices were associated with fatty liver [14], albuminuria, advanced kidney disease [15], lung function [16], osteoporosis [17], hypertension [18], peripheral artery disease [19], and dementia [20]. Moreover, although both overweight and underweight have been associated with osteoporosis [21], few studies have explored the relationship between osteoporosis and indices related to obesity. The relationship between BMI and the risk of fracture has been demonstrated to be inverse and nonlinear, with the greatest risk in subjects with BMI < 20 kg/m^2^ and only a small risk reduction in those with a BMI > 25 kg/m^2^ [22,23]. Another systematic review and meta-analysis indicated that adult obese patients, defined as BMI > 30 kg/m^2^ or >28 kg/m^2^ in China, had higher lumbar spine and femoral neck BMD than patients with a healthy weight [24]. A systematic review found that low BMI (20–25 kg/m^2^) was an important and modifiable risk factor for the development of osteoporosis and osteoporotic fractures [25]. Therefore, this longitudinal study aimed to investigate the relationships of indices related to obesity with baseline and change in calcaneus ultrasound T-score (ΔT-score).

## 2. Materials and Methods

### 2.1. Taiwan Biobank

**Figure 1:**
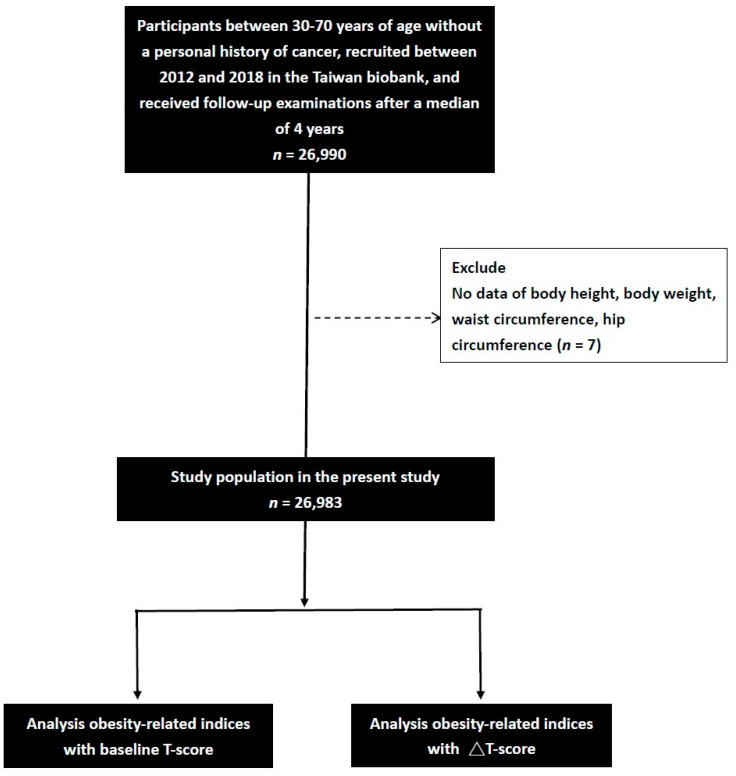
Flowchart of study population. The change in T-score (ΔT-score) was calculated as the T-score at follow-up minus that at baseline.

A total of 26,990 enrollees in the Taiwan Biobank had a median of 4 years of follow-up data. Those with no data on WC (*n* = 1), HC (*n* = 1), BH (*n* = 1), and BW (*n* = 4) were excluded, and a total of 26,983 participants (mean age 51.2 ± 10.4 years; men: 9543; women: 17,440) were included for analysis (Figure 1).

The Taiwan Biobank was launched by the Taiwan government in 2012 as a prospective study of citizens aged 30–70 recruited from centers around Taiwan for epidemiological and biomedical research purposes. For each enrollee in the Taiwan Biobank, comprehensive genomic and phenotypic data are collected and recorded at enrolment and during follow-up through urine and blood tests (for uric acid, fasting glucose, hemoglobin, TG, total/HDL/low-density lipoprotein (LDL) cholesterol), physical examination (for BH, BW, HC, and WC), and structured questionnaires (for age, sex, and medical history of hypertension and diabetes mellitus (DM)) [26,27]. Ethical approval for the Taiwan Biobank was given by the Ethics and Governance Council of the Taiwan Biobank and Institutional Review Board on Biomedical Science Research, Academia Sinica, Taiwan. All participants in the Taiwan Biobank provided written informed consent before enrollment.

Body mass index was calculated as BW/BH^2^, and the estimated glomerular filtration rate (eGFR) was calculated using the 4-variable MDRD equation [28]. Trained personnel performed blood pressure (BP) measurements digitally with the participants abstaining from exercise, caffeine, and smoking for a minimum of 30 min before measurements. All measurements were taken three times with a 1–2 min break between measurements, with average values being used for analysis. The “Physical Fitness 333 Plan” criteria were used to define regular exercise, as promoted by the Ministry of Education in Taiwan, as at least 30 min three times a week [29]. 

This study was conducted according to the Declaration of Helsinki and approved by the Institutional Review Board of Kaohsiung Medical University Hospital (KMUHIRB-E(I)-20210058) on 8 April 2021.

### 2.2. Assessment of Calcaneus Ultrasound T-Score

The calcaneus of the non-dominant foot was used for ultrasound measurements (Achilles InSight, GE, Madison Heights, MI, USA) to calculate the T-score as (the participant’s T-score minus the mean T-score in a normal young adult population) divided by the SD of the normal young-adult population. The change in T-score (ΔT-score) was calculated as the T-score at follow-up minus that at baseline.

### 2.3. Calculation of Obesity-Related Indices

The calculation of obesity-related indices is shown in Table 1.

### 2.4. Statistical Analysis

Data are shown as number (%) and mean (±SD), as appropriate. The chi-square and independent t tests were used to examine differences in categorical and continuous variables, respectively. Significant variables in univariable analysis were used for multivariable analysis, which was used to evaluate the association of each obesity-related index with baseline T-score and ΔT-score. Results were considered significant at *p* < 0.05. SPSS version 19.0 for Windows (IBM Corp., Armonk, NY, USA) was used for the statistical analysis. 

## 3. Results

The 26,983 enrolled participants were divided into two groups by baseline calcaneus T-score: ≥ −2.5 (*n* = 24,932; 92.4%) and <−2.5 (*n* = 2051; 7.6%). 

### 3.1. Clinical Characteristics of the Study Groups 

The T-score < −2.5 group had a higher prevalence of hypertension, regular exercise, and smoking, were older, predominantly male, and had higher systolic and diastolic BPs, higher menstruation status, higher BW and HC, lower T-scores at follow-up and baseline, higher fasting glucose and hemoglobin, lower eGFR, BMI, and BAI, and higher WHR compared to the T-score ≥ −2.5 group (Table 2). 

### 3.2. Factors Associated with Baseline Calcaneus T-Score Using Univariable Analysis 

Univariable linear regression analysis showed that a low baseline T-score was significantly associated with male sex, hypertension, regular exercise, older age, DM, smoking, drinking alcohol, high systolic and diastolic BPs, menopause, high hemoglobin, LDL-C, TG, uric acid, fasting glucose and total cholesterol, and low HDL-C and eGFR (Table 3).

### 3.3. Multivariable Analysis to Analyze the Relationships between the Indices Related to Obesity and Baseline Calcaneus Ultrasound T-Score

The following multivariable linear regression analysis models were conducted to determine relationships among the indices related to obesity with baseline T-score (Table 4).

The analysis showed that a low baseline T-score was significantly associated with low values of BMI (per 1 kg/m^2^; unstandardized coefficient (β), 0.065; 95% confidence interval (CI), 0.058 to 0.072; *p* < 0.001), WHR (per 1%; β, 0.012; 95% CI, 0.008 to 0.016; *p* < 0.001), WHtR (per 1%; β, 0.024; 95% CI, 0.020 to 0.029; *p* < 0.001), BRI (per 1; β, 0.079; 95% CI, 0.066 to 0.093; *p* < 0.001), BAI (per 1; β, 0.032; 95% CI, 0.025 to 0.038; *p* < 0.001), AVI (per 1; β, 0.049; 95% CI, 0.041 to 0.057; *p* < 0.001), and LAP (per 1; β, 0.005; 95% CI, 0.004 to 0.0064; *p* = 0.022). However, no association between VAI and baseline T-score was found.

### 3.4. Factors Associated with ΔT-Score Using Univariable Analysis 

Univariable linear regression analysis showed that a low ΔT-score was significantly associated with uric acid, female sex, older age, low diastolic BP, regular exercise, no alcohol or smoking history, menopause, hemoglobin, low fasting glucose, TG, and total/HDL-C (Table 5).

### 3.5. Relationships among the Indices Related to Obesity with ΔT-Score in Multivariable Analysis

The following multivariable linear regression analysis models were conducted to determine relationships among the indices related to obesity with T-score (Table 6).

The analysis showed that a low ΔT-score was significantly associated with low values of BMI (per 1 kg/m^2^; β, 0.005; 95% CI, 0 to 0.011; *p = 0.036*), BAI (per 1; β, 0.010; 95% CI, 0.005 to 0.014; *p* < 0.001), and VAI (per 1; β, 0.017; 95% CI, 0.006 to 0.027; *p* = 0.002). However, no associations between LAP, WHR, WHtR, BRI, or AVI with ΔT-score were found.

## 4. Discussion

The results of this study showed that low LAP, AVI, BAI, BMI, BRI, WHR, and WHtR were associated with low baseline T-score. In addition, we found significant associations between a low ΔT-score with low BMI, BAI, and VAI.

An interesting finding was that a low baseline T-score was associated with low values of all the indices related to obesity studied except VAI. Low weight is a well-known risk factor for the future, whereas a high BMI has been suggested to have a protective effect [35,36,37,38,39,40]. From a public health point of view, however, the story is more complicated. Obesity is associated with increased morbidity from diabetes, hypertension, and cardiovascular diseases, and is also associated with increased mortality [23,41]. It is important, therefore, to quantify the association between BMI and fracture risk and to explore its relationship to age, sex, and BMD with the aim of being able to give balanced advice on lifestyle to patients. A previous meta-analysis found that without information on BMD, a decreased BMI in both women and men was correlated with a significantly higher age-specific fracture risk, and that those with increased BMI values had a decreased fracture risk [23]. However, when including information on BMD, the risk ratio per unit increase in BMI was found to change remarkably and to remain significantly different only for hip fractures in women [23]. Obesity has been shown to have a protective effect against fractures at some locations [22,42]. For example, obese postmenopausal women and men have been reported to have significantly reduced risks of fractures of the hip, pelvis, and wrist [43]. Another report from Catalonia also reported an association between obesity with lower risks of spine and hip fractures [44]. For osteoporotic fracture, a high BMI had a greater and significant protective effect in the absence of BMD [42]. With regard to health and mortality, WC and WHR are indicators of central adiposity and negatively impact both regardless of BMI [45,46]. The WC and WHR have been suggested by the World Health Organization as useful indicators of illness risk [47]. In addition, a cross-sectional study from Egypt reported a positive correlation between bone health and BMI, fat mass, and its distribution and basal metabolic rate, especially at the femoral neck, in women pre- and post-menopause, suggesting that overweight/obesity plays a protective role in bone health [48]. In a Chinese study, 5.75% of 9135 male participants had osteoporosis, among whom large body mass, including BMI, BRI, WHtR, WHR, a body shape index, and WC, was negatively associated with osteoporosis in older and middle-aged groups, and BMI was the strongest predictor of osteoporosis [49]. Moreover, a cross-sectional study of the *United Kingdom* Biobank including 3674 participants with magnetic resonance imaging and DXA findings found positive associations among abdominal subcutaneous adipose tissue, visceral adipose tissue, and total body adiposity with BMD [50]. A possible explanation for the association of obesity with high BMD is that obesity may cause an increase in BMD due to an elevated 17β-estradiol level with increased mechanical load, which may have a protective effect on bone [51]. Other studies have suggested that increased daily protein intake could positively affect BMD and functional performance [52,53,54,55]. 

We also found that low BMI, BAI, and VAI were significantly correlated with low ΔT-score, indicating a rapid decline in T-score. Several studies have reported greater bone loss in elderly underweight subjects compared to control subjects, with T-score values below −2.5 [56,57]. The mechanisms underlying this condition could be muscle weakness brought on by protein or vitamin D nutritional deficits, a loss of cushioning over the greater trochanter, or an increased risk of falling [58,59,60]. Underweight individuals have been shown to be at an increased risk of malnutrition [56]. Malnutrition, in particular with regard to protein, has been correlated with a higher risk of fractures related to osteoporosis through a decrease in bone mass affecting muscle strength [61]. Malnutrition, physical inactivity, and various other factors can cause tissue loss, a condition known as sarcopenia. Etiological factors include certain cytokines which can affect bone restructuring and proteolysis, and subsequently osteoclast metabolism. Mechanisms resulting in the release of cytokines such as inflammation and stress have been associated with unfavorable restructuring of bone and protein mass loss, resulting in worse muscular function prognosis [62,63,64]. Further studies are needed to elucidate the etiology; however, it may be partially due to the acceleration of bone mineral loss with repeated fracture episodes, which is more likely to occur in those who are underweight due to a decrease in muscle strength, malnutrition, and low BMD [21]. Weight bearing has been shown to enhance bone density through an effect on cells [65], and animal studies have shown that osteocytes are particularly sensitive to biomechanical stress [66]. Several reports have shown that osteoclasts underwent apoptosis in the absence of loading, but that this process was inhibited if the osteoclasts detected shear stress signals [67,68]. At the same time, osteoclast activity was inhibited and osteoblast differentiation was promoted. These findings are compatible with the present study, in that low values of the obesity-related indices, which would cause lower loading on osteocytes by weight bearing, were associated with low BMD and a rapid decline in T-score. These findings have important implications for the identification of possible cases based on clinical risk factors, and they suggest that avoiding being underweight may be important in helping to prevent osteoporosis in Taiwan. 

Despite the inclusion of a large participant cohort with complete follow-up data, some limitations should be mentioned. First, we did not have prescription data for the participants, and the prevention and development of osteoporosis could have been affected by some medications. Second, we did not use DXA to confirm the presence of osteoporosis but rather ultrasound. The current diagnostic criteria are based on BMD, which is most commonly measured using DXA. Nevertheless, use of the T-score is recommended in research of osteoporosis pharmaceutical interventions. Moreover, ultrasound is relatively cost-efficient and can avoid radiation exposure. Furthermore, a previous study on women in China reported that the ultrasound system used in this study could identify osteoporosis as determined by axial BMD on DXA [69]. Another limitation is that as only approximately half of the Taiwan Biobank enrollees returned for follow-up examinations, sample bias cannot be ruled out, and this may have affected our findings. Finally, further studies may be necessary to verify our findings in other ethnicities, as all enrollees in the Taiwan Biobank are ethnically Chinese.

## 5. Conclusions

Low values of all indices related to obesity analyzed in this study except VAI were associated with a low baseline T-score. Furthermore, a low ΔT-score, representing a rapid decrease in T-score, was associated with low BMI, BAI, and VAI. Hence, avoiding being underweight may be important in helping to prevent osteoporosis in Taiwan.

## Figures and Tables

**Table 1:** Calculation formula of each obesity-related index.

Item	Calculation Formula
BMI	BW (kg)/BH^2^ (m)
WHR	WC (cm)/HC (cm)
WHtR	WC (cm)/BH (cm)
BRI	$364.2-365.5\times \sqrt{1-{\left(\frac{\frac{{\mathrm{WC}}_{\left(m\right)}}{2\pi }}{0.5\times {\mathrm{BH}}_{\left(m\right)}}\right)}^{2}\;\;}$ [30]
BAI	$\frac{{\mathrm{HC}}_{\left(\mathrm{cm}\right)}}{{\mathrm{BH}}_{\left(m\right)}{}^{3/2}}-18\;$ [31]
AVI	$\frac{2\times {\left({\mathrm{WC}}_{\left(\mathrm{cm}\right)}\right)}^{2}+0.7\times {\left({\mathrm{WC}}_{\left(\mathrm{cm}\right)}-{\mathrm{HC}\;}_{\left(\mathrm{cm}\right)}\right)}^{2}}{1000}\;$ [32]
LAP	$\left({\mathrm{WC}}_{\left(\mathrm{cm}\right)}-65\right)\times {\;\mathrm{TG}}_{\left(\mathrm{mmol}/L\right)}$ in males, and $\left({\mathrm{WC}}_{\left(\mathrm{cm}\right)}-58\right)\times {\;\mathrm{TG}}_{\left(\mathrm{mmol}/L\right)}$ in females [33]
VAI	$\left(\frac{{\mathrm{WC}}_{\left(\mathrm{cm}\right)}}{39.68+\left(1.88\times \;\mathrm{BMI}\right)}\right)\times \left(\frac{{\mathrm{TG}}_{\left(\mathrm{mmol}/L\right)}}{1.03}\right)\times \left(\frac{1.31}{\mathrm{HDL}-C_{\left(\mathrm{mmol}/L\right)}}\right)$ in males, and $\left(\frac{{\mathrm{WC}}_{\left(\mathrm{cm}\right)}}{36.58+\left(1.89\times \;\mathrm{BMI}\right)}\right)\times \left(\frac{{\mathrm{TG}}_{\left(\mathrm{mmol}/L\right)}}{0.81}\right)\times \left(\frac{1.52}{\mathrm{HDL}-C_{\left(\mathrm{mmol}/L\right)}}\right)$ in females [34]

Abbreviations: BMI—body mass index; WHR—waist-to-hip ratio; WHtR—waist-to-height ratio; BRI—body roundness index; BAI—body adiposity index; AVI—abdominal volume index; LAP—lipid accumulation product; VAI—visceral adiposity index.

**Table 2:** Comparison of clinical characteristics among participants according to T score ≥ −2.5 or <−2.5.

Characteristics	T-Score ≥ −2.5 (*n* = 24,932)	T-Score < −2.5 (*n* = 2051)	*p*
Age (year)	50.7 ± 10.4	57.2 ± 8.4	<0.001
Male gender (%)	34.8	41.8	<0.001
DM (%)	5.2	6.0	0.119
Hypertension (%)	12.8	16.7	<0.001
Systolic BP (mmHg)	117.2 ± 17.6	121.5 ± 18.3	<0.001
Diastolic BP (mmHg)	72.4 ± 10.8	73.2 ± 10.9	0.002
Smoking history (%)	25.3	29.5	<0.001
Alcohol history (%)	2.9	3.2	0.427
Regular exercise habit (%)	47.8	54.3	<0.001
Menstruation in female (%)	47.3	10.9	<0.001
Body height (cm)	161.1 ± 8.0	161.0 ± 8.7	0.416
Body weight (Kg)	62.9 ± 11.8	61.2 ± 12.1	<0.001
Waist circumference (cm)	83.2 ± 9.8	83.0 ± 9.9	0.415
Hip circumference (cm)	95.9 ± 6.8	94.7 ± 6.7	<0.001
Baseline T-score	−0.265 ± 1.472	−3.073 ± 0.499	<0.001
Follow-up T-score	−0.571 ± 1.482	−2.832 ± 1.067	<0.001
Laboratory parameters			
Fasting glucose (mg/dL)	96.1 ± 20.0	98.0 ± 23.5	<0.001
Hemoglobin (g/dL)	13.7 ± 1.6	13.8 ± 1.4	0.045
Triglyceride (mg/dL)	114.0 ± 83.9	113.5 ± 72.3	0.770
Total cholesterol (mg/dL)	195.3 ± 35.4	196.8 ± 35.8	0.062
HDL-C (mg/dL)	54.2 ± 13.2	54.5 ± 13.6	0.464
LDL-C (mg/dL)	121.6 ± 31.7	121.7 ± 31.0	0.942
eGFR (mL/min/1.73 m^2^)	109.3 ± 25.3	107.5 ± 26.5	<0.001
Uric acid (mg/dL)	5.5 ± 1.4	5.5 ± 1.4	0.961
Obesity-related indices			
BMI (kg/m^2^)	24.1 ± 3.6	23.5 ± 3.6	<0.001
WHR (%)	86.7 ± 6.8	87.5 ± 6.8	<0.001
WHtR (%)	51.7 ± 6.0	51.6 ± 6.0	0.685
BRI	6.8 ± 1.9	6.7 ± 1.9	0.559
BAI	29.0 ± 4.1	28.5 ± 4.1	<0.001
AVI	14.2 ± 3.3	14.1 ± 3.3	0.332
LAP	31.5 ± 30.8	30.3 ± 27.5	0.097
VAI	1.69 ± 1.67	1.66 ± 1.45	0.353

Abbreviations. DM—diabetes mellitus; BP—blood pressure; HDL-C—high-density lipoprotein cholesterol; LDL-C—low-density lipoprotein cholesterol; eGFR—estimated glomerular filtration rate; BMI—body mass index; WHR—waist–hip ratio; WHtR—waist-to-height ratio; BRI—body roundness index; BAI—body adiposity index; AVI—abdominal volume index; LAP—lipid accumulation product; VAI—visceral adiposity index.

**Table 3:** Determinants of baseline T-score using univariable linear regression analysis.

Parameters	Baseline T-Score		
	Univariable		
	Unstandardized Coefficient β	95% CI	*p*
Age (per 1 year)	−0.053	−0.055, −0.051	<0.001
Female (vs. male)	0.432	0.392, 0.472	<0.001
DM	−0.267	−0.353, −0.182	<0.001
Hypertension	−0.416	−0.472, −0.359	<0.001
Systolic BP (per 1 mmHg)	−0.014	−0.015, −0.012	<0.001
Diastolic BP (per 1 mmHg)	−0.012	−0.014, −0.010	<0.001
Smoking history	−0.272	−0.315, −0.228	<0.001
Alcohol history	−0.162	−0.276, −0.047	0.006
Regular exercise habits	−0.218	−0.256, −0.179	<0.001
Menstruation in female	1.371	1.325, 1.416	<0.001
Laboratory parameters			
Fasting glucose (per 1 mg/dL)	−0.006	−0.007, −0.005	<0.001
Hemoglobin (per 1 g/dL)	−0.086	−0.098. −0.074	<0.001
Triglyceride (per 1 mg/dL)	−0.001	−0.001, −0.001	<0.001
Total cholesterol (per 1 mg/dL)	−0.003	−0.004, −0.003	<0.001
HDL-cholesterol (per 1 mg/dL)	0.005	0.003, 0.006	<0.001
LDL-cholesterol (per 1 mg/dL)	−0.003	−0.003, −0.002	<0.001
eGFR (per 1 mL/min/1.73 m^2^)	0.005	0.005, 0.006	<0.001
Uric acid (per 1 mg/dL)	−0.072	−0.086, −0.059	<0.001

Values expressed as unstandardized coefficient β and 95% confidence interval (CI). Abbreviations are the same as in Table 1.

**Table 4:** Determinants of baseline T-score using multivariable linear regression analysis.

Obesity-Related Indices	Baseline T-Score		
	Multivariable		
	Unstandardized Coefficient β	95% CI	*p*
BMI (per 1 kg/m^2^) ^a^	0.065	0.058, 0.072	<0.001
WHR (per 1%) ^a^	0.012	0.008, 0.016	<0.001
WHtR (per 1%) ^a^	0.024	0.020, 0.029	<0.001
BRI (per 1) ^a^	0.079	0.066, 0.093	<0.001
BAI (per 1) ^a^	0.032	0.025, 0.038	<0.001
AVI (per 1) ^a^	0.049	0.041, 0.057	<0.001
LAP (per 1) ^b^	0.005	0.004, 0.006	<0.001
VAI (per 1) ^c^	0.003	−0.012, 0.019	0.667

Values expressed as unstandardized coefficient β and 95% confidence interval (CI). Abbreviations are the same as in Table 1. ^a^ Covariates in the multivariable model included age, sex, diabetes, hypertension, systolic and diastolic BPs, smoking and alcohol history, regular exercise, menstruation status in female, fasting glucose, hemoglobin, TGs, total cholesterol, HDL-C, LDL-C, eGFR, and uric acid (significant variables in Table 2). ^b^ Covariates as ^a^ Covariates, except for triglyceride. ^c^ Covariates as ^a^ Covariates, except for triglyceride and HDL-C.

**Table 5:** Determinants of ΔT-score using univariable linear regression analysis.

Parameters	ΔT-Score		
	Univariable		
	Unstandardized Coefficient β	95% CI	*p*
Age (per 1 year)	−0.005	−0.006, −0.004	<0.001
Female (vs. male)	−0.160	−0.185, −0.135	<0.001
DM	0.035	−0.018, 0.089	0.194
Hypertension	0.024	−0.012, 0.059	0.186
Systolic BP (per 1 mmHg)	−7.13 × 10^−5^	−0.001, 0.001	0.836
Diastolic BP (per 1 mmHg)	0.002	0.001, 0.003	<0.001
Smoking history	0.111	0.084, 0.138	<0.001
Alcohol history	0.110	0.038, 0.181	0.003
Regular exercise habits	−0.036	−0.059, −0.012	0.004
Menstruation in female	0.122	0.090, 0.153	<0.001
Laboratory parameters			
Fasting glucose (per 1 mg/dL)	0.001	0, 0.002	0.001
Hemoglobin (per 1 g/dL)	0.019	0.012, 0.027	<0.001
Triglyceride (per 1 mg/dL)	0	0, 0	0.003
Total cholesterol (per 1 mg/dL)	−0.001	−0.001, −0.001	<0.001
HDL-cholesterol (per 1 mg/dL)	−0.005	−0.006, −0.004	<0.001
LDL-cholesterol (per 1 mg/dL)	0	−0.001, 0	0.105
eGFR (per 1 mL/min/1.73 m^2^)	−1.142 × 10^−5^	0, 0	0.962
Uric acid (per 1 mg/dL)	0.031	0.023, 0.040	<0.001

Values expressed as unstandardized coefficient β and 95% confidence interval (CI). Abbreviations are the same as in Table 1. The change in T-score (ΔT-score) was calculated as the T-score at follow-up minus that at baseline.

**Table 6:** Determinants of ΔT-score using multivariable linear regression analysis.

Obesity-Related Indices	ΔT-Score		
	Multivariable		
	Unstandardized Coefficient β	95% CI	*p*
BMI (per 1 kg/m^2^) ^a^	0.005	0, 0.011	0.036
WHR (per 1%) ^a^	−0.002	−0.005, 0.001	0.153
WHtR (per 1%) ^a^	0.003	0, 0.006	0.076
BRI (per 1) ^a^	0.007	−0.002, 0.017	0.122
BAI (per 1) ^a^	0.010	0.005, 0.014	<0.001
AVI (per 1) ^a^	0.003	−0.002, 0.009	0.233
LAP (per 1) ^b^	0	−0.001, 0.001	0.728
VAI (per 1) ^c^	0.017	0.006, 0.027	0.002

Values expressed as unstandardized coefficient β and 95% confidence interval (CI). Abbreviations are the same as in Table 1. ^a^ Covariates in the multivariable model included age, sex, diastolic BP, smoking and alcohol history, regular exercise, menstruation status in female, fasting glucose, hemoglobin, TGs, total cholesterol, HDL-C, and uric acid (significant variables in Table 4). ^b^ Covariates as ^a^ Covariates, except for triglyceride. ^c^ Covariates as ^a^ Covariates, except for triglyceride and HDL-C.

## Data Availability

The data underlying this study are from the Taiwan Biobank. Due to restrictions placed on the data by the Personal Information Protection Act of Taiwan, the minimal data set cannot be made publicly available. Data may be available upon request to interested researchers. Please send data requests to: Szu-Chia Chen, PhD, MD. Division of Nephrology, Department of Internal Medicine, Kaohsiung Medical University Hospital, Kaohsiung Medical University.
